# The Multidisciplinary Management of Cutaneous Squamous Cell Carcinoma: A Comprehensive Review and Clinical Recommendations by a Panel of Experts

**DOI:** 10.3390/cancers14020377

**Published:** 2022-01-13

**Authors:** Ignazio Stanganelli, Francesco Spagnolo, Giuseppe Argenziano, Paolo A. Ascierto, Franco Bassetto, Paolo Bossi, Vittorio Donato, Daniela Massi, Cesare Massone, Roberto Patuzzo, Giovanni Pellacani, Pietro Quaglino, Paola Queirolo, Iris Zalaudek, Giuseppe Palmieri, on behalf of Italian Melanoma Intergroup (IMI)

**Affiliations:** 1 Skin Cancer Unit, Scientific Institute of Romagna for the Study of Cancer, IRCSS IRST, 47014 Meldola, Italy; ignazio.stanganelli@irst.emr.it; 2 Department of Dermatology, University of Parma, 43121 Parma, Italy; 3 Deparment of Medical Oncology, IRCCS Ospedale Policlinico San Martino, 16132 Genova, Italy; 4 Dermatology Unit, University of Campania Luigi Vanvitelli, 80138 Naples, Italy; giuseppe.argenziano@unicampania.it; 5 Department of Melanoma, Cancer Immunotherapy and Development Therapeutics, Istituto Nazionale Tumori IRCCS “Fondazione G. Pascale”, 80131 Naples, Italy; p.ascierto@istitutotumori.na.it; 6 Clinic of Plastic and Reconstructive Surgery, Padova University-Hospital, 35128 Padova, Italy; franco.bassetto@unipd.it; 7 Medical Oncology Unit, Department of Medical and Surgical Specialties, Radiological Sciences and Public Health, ASST Spedali Civili of Brescia, University of Brescia, 25123 Brescia, Italy; paolo.bossi@unibs.it; 8 Radiation Oncology Department, Azienda Ospedaliera San Camillo-Forlanini, 00152 Rome, Italy; vdonato@scamilloforlanini.rm.it; 9 Section of Anatomic Pathology, Department of Health Sciences, University of Florence, 50134 Florence, Italy; daniela.massi@unifi.it; 10 Dermatology Unit, Galliera Hospital, 16128 Genoa, Italy; cesare.massone@galliera.it; 11 Melanoma and Sarcoma Unit, Department of Surgery, IRCCS Fondazione Istituto Nazionale dei Tumori, 20133 Milan, Italy; roberto.patuzzo@istitutotumori.mi.it; 12 Department of Dermatology, University of Modena and Reggio Emilia, 41124 Modena, Italy; giovanni.pellacani@unimore.it; 13 Dermatology Unit, Department of Medical Sciences, University of Turin, 10126 Turin, Italy; pietro.quaglino@unito.it; 14 Division of Medical Oncology for Melanoma, Sarcoma and Rare Tumors, European Institute of Oncology IRCCS, 20141 Milan, Italy; paola.queirolo@ieo.it; 15 Dermatology and Venereology Department, Ospedale Maggiore di Trieste, University of Trieste, 34125 Trieste, Italy; iris.zalaudek@asuits.sanita.fvg.it; 16 Unit of Cancer Genetics, Institute of Genetic and Biomedical Research (IRGB), National Research Council (CNR), 07100 Sassari, Italy; giuseppe.palmieri@cnr.it

**Keywords:** cutaneous squamous cell carcinoma, keratinocyte carcinomas, skin cancer, guidelines, recommendations, cemiplimab, immunotherapy, anti-PD-1, immune checkpoint inhibitors

## Abstract

**Simple Summary:**

Cutaneous squamous cell carcinoma is one of the most common forms of cancer. Although most cases are cured with surgical excision, a few tumors are associated with a high risk of local or distant relapse; therefore, it is relevant to identify high-risk lesions among all other low-risk CSCCs for the proper diagnostic and therapeutic management. Chemotherapy achieves mostly short-lived responses that do not lead to a curative effect and are associated with severe toxicities. Recently, PD-1 inhibitor cemiplimab was approved by the regulatory authorities for the treatment of advanced cutaneous squamous cell carcinoma; subsequently, the anti-PD-1 agent pembrolizumab received the approval by the FDA only in the same setting. Here, we provide a literature review and clinical recommendations by a panel of experts regarding the diagnosis, treatment, and follow-up of cutaneous squamous cell carcinoma.

**Abstract:**

Cutaneous squamous cell carcinomas (CSCC) account for about 20% of all keratinocyte carcinomas, which are the most common form of cancer. Heterogeneity of treatments and low mortality are a challenge in obtaining accurate incidence data and consistent registration in cancer registries. Indeed, CSCC mostly presents as an indolent, low-risk lesion, with five-year cure rates greater than 90% after surgical excision, and only few tumors are associated with a high-risk of local or distant relapse; therefore, it is particularly relevant to identify high-risk lesions among all other low-risk CSCCs for the proper diagnostic and therapeutic management. Chemotherapy achieves mostly short-lived responses that do not lead to a curative effect and are associated with severe toxicities. Due to an etiopathogenesis largely relying on chronic UV radiation exposure, CSCC is among the tumors with the highest rate of somatic mutations, which are associated with increased response rates to immunotherapy. Thanks to such strong pre-clinical rationale, clinical trials led to the approval of anti-PD-1 cemiplimab by the FDA (Food and Drug Administration) and EMA (European Medicines Agency), and anti-PD-1 pembrolizumab by the FDA only. Here, we provide a literature review and clinical recommendations by a panel of experts regarding the diagnosis, treatment, and follow-up of CSCC.

## 1. Methodology and Scopes of the Clinical Recommendations

A consensus web meeting was held on 24 September 2020 with the objective of discussing and reporting expert clinical recommendations on the management of cutaneous squamous cell carcinoma (CSCC). The main topics covered by these recommendations are clinical and histopathological diagnosis of CSCC, surgical treatment of primary tumors, the role of radiotherapy, follow-up, and the role of immunotherapy for the treatment of locally advanced and metastatic disease. As compared with previous publications, the aim of the panel of experts was to extend the focus of the recommendations to cover other topics such as data collection in cancer registries, use of molecular biomarkers for patient risk stratification, management of field cancerization, and use of immunotherapy in special populations (patients with autoimmune disease requiring systemic immunosuppression, with a history of solid organ transplant, with an HIV/HBV/HCV infection, and with hematologic malignancies). Following the web meeting, the draft of the manuscript was conducted through emailing among the panel of experts.

## 2. Epidemiology

Cutaneous squamous cell carcinoma is a form of keratinocyte carcinoma and includes a heterogeneous group of cancers presenting differences in terms of morphology, growth kinetics, biology, and metastatic potential; they are the second most common form of skin cancer after basal cell carcinomas, accounting for about 20% of all keratinocyte carcinomas [1,2]. Rates of keratinocyte carcinomas are increasing [3,4] and are predicted to continue to increase [5]. The highest frequency of keratinocyte carcinomas is registered in Australia, with an estimated age-standardized incidence of CSCC as high as 387 cases per 100.000 individuals [6]. In the United States (US), CSCC is not included in the national tumor registries, but data from the Rochester Epidemiology Project, conducted by the Mayo Clinic, showed an overall 263% increase in the incidence of CSCC between the 1976 to 1984 and 2000 to 2010 periods [7]. Over 700.000 new cases of CSCC are estimated to be diagnosed each year in the United States, and about 3900–8800 people are estimated to die annually due to CSCC [8]. In Europe, the age-standardized incidence of CSCC is highly variable, ranging from 9 to 96 per 100,000 for male individuals and 5 to 68 per 100,000 for females [9,10,11,12]. Such variability may be due not only to a phenotypic variability among different European populations, but also to a heterogeneous registration of CSCC cases in national registries [12].

As in the United States and most European countries, CSCC incidence data from a unified national registry are not available in Italy. In the AIRTUM 2019 report, 19,000 new cases of CSCC were estimated for year 2018, with an overall estimated incidence of about 19 per 100.000 inhabitants, and a North–South gradient (higher incidence in northern and lower incidence in southern Italy) [13].

Heterogeneity of treatments (including non-surgical ones) and low mortality are a challenge in obtaining accurate incidence data and consistent registration in cancer registries. Important consequences are that the public health burden and social costs associated with CSCC are probably underestimated, and that studies on the prognostic features of CSCC are difficult to conduct. For these reasons, all CSCC cases, including in situ lesions, should be included in cancer registries, with both tumor- and patient-related information, to ensure consistent collection of information and to facilitate analysis of data (expert recommendation 1). To ensure homogeneity of data collection, all pathologists should adhere to the WHO classification of Skin Tumours 4th edition (expert recommendation 2) [14]. According to this classifications, actinic keratoses are not classified as CSCC in situ [14].

Overall, the chance for long-term survival is high, and only in a very small subset of patients with more aggressive lesions locoregional and/or distant metastases may occur [15]. Advanced CSCC includes locally advanced CSCC (laCSCC), i.e., not amenable to either surgery or radiotherapy with a reasonable curative intent, and metastatic CSCC (mCSCC), which includes cases with regional and/or distant metastases [16]. In a study reporting on more than 900 patients with CSCC, who were followed-up for about 10 years, relapse occurred in only 4.6% of cases. Lymph nodes metastases occurred in 3.7% of patients, while 2.1% of patients died due to progressive disease. In case of metastases, loco-regional lymph nodes were involved in the 85% of patients, while distant metastases were more frequently located in the lungs, liver, brain, skin, and bones. The risk of relapse was associated with some tumor-related characteristics, such as tumor diameter of at least 2 cm, poor differentiation, invasion beyond fat, and perineural invasion [17].

## 3. Risk Factors

Most CSCCs originate in chronically sun-damaged skin. In fact, ultraviolet (UV) radiation exposure is by far the most important causal factor for CSCC, followed by age, fair skin, and immunosuppression. The kind of UV exposure that is most frequently associated with CSCC is chronic, cumulative exposure. In about 90% of cases, CSCC originates in chronically exposed anatomical areas, such as head and neck, or dorsum of the hands and forearms; CSCC is more common in outdoor workers. Therefore, CSCC should be considered as an occupational disease in a subset of outside workers at high risk of developing skin cancer and be recorded in specific registries to gain access to specific welfare benefits (expert recommendation 3). Additional risk factors include artificial UV radiation, such as PUVA therapy and indoor tanning devices, which are associated with increased risk of CSCC, especially for individuals who are exposed at an age <25 years. Avoiding an excessive exposure to UV radiation is the most relevant form of primary prevention of CSCC [18].

Immunosuppression is another relevant factor for both risk of developing primary CSCCs and increased risk of relapse in patients with previous diagnosis of CSCC. Immunosuppressed patients mainly include those with human immunodeficiency virus (HIV) infection, those who received a solid organ transplant or hematopoietic stem cell transplant, and those with chronic lymphocytic leukemia (CLL) [19,20].

Finally, other conditions associated with an increased risk of CSCC are some hereditary syndromes such as xeroderma pigmentosum, epidermolysis bullosa, oculocutaneous albinism and Fanconi anaemia, and Lynch syndrome/Muir Torre syndrome [1].

## 4. Pathogenesis and Molecular Characteristics

As a result of chronic UV radiation exposure, CSCCs harbor a UV mutation signature, characterized by a prevalence of C > T or CC > TT dinucleotide mutations, and a mutation rate as high as about 50 mutations per megabase DNA pair, which is higher than most solid tumors [21]. Genes altered in UV-induced CSCC include CDKN2A, TP53, MC1R, XPC, PTCH1, telomerase, CYP2D6, and GsTT1, while genetic alterations that may be targeted with treatments, such as BRAF and epidermal growth factor receptor (EGFR), are infrequent [22]. Genetic signatures have been linked to tumor differentiation (well-differentiated and moderately/poorly differentiated) [21]. In a study on 23 biopsies of 23 patients with CSCC at different stages (from premalignant actinic keratosis to low-risk invasive and high-risk non-metastatic and mCSCC), genome-wide DNA methylation profiling identified specific epigenetic features in high-risk tumors. Moreover, a prognostic prediction model identified a methylation signature able to predict OS [23]. Nevertheless, it appeared that epigenetic deregulation was not a sequential continuum from AK to mCSCC, and that, on the contrary, the methylation signature of AKs was similar to that of mCSCC, and different from that of initial invasive carcinomas and high-risk non-metastatic carcinomas [23].

Although most CSCC have good prognosis and are successfully treated with surgical excision, a subgroup of patients with high-risk CSCC may relapse and die due to locally advanced or metastatic disease. Contemporary staging systems, such as the AJCC and the Brigham and Women’s Hospital (BWH) classifications, as well as risk factors identified by guidelines, such as the NCCN clinical recommendations, are limited in identifying with accuracy patients who are at high risk of metastasis [24]. Therefore, the identification of molecular and epigenetic risk factors associated with these aggressive subtypes is of major interest and is currently an unmet need, despite some promising advances having been made in the last years. In a series of CSCCs, telomerase reverse transcriptase gene (*TERT*) promoter mutations were independently associated with risk of lymph node metastasis, with an odds ratio of 15.89 [25]. Loss of inositol polyphosphate-5-phosphatase (INPP5A) was also found to play an important role into risk of metastatic progression of primary CSCC (hazard ratio: 4.71) and to predict poor survival of recurrent and metastatic CSCC, with an overall survival of 31.0 months for patients with low expression of INPP5A as compared with 62.0 months for high-expression cases [26,27]. More recently, a 40-gene expression profile test was developed and validated in a large cohort of 586 primary CSCC samples from 23 independent centers to predict the risk of metastasis in localized CSCC with at least one NCCN high-risk feature. This 40-gene expression profile was able to stratify patients with high-risk CSCC into three classes on the basis of the risk of developing metastasis within 3 years from diagnosis. Patients with low risk (class 1) and high risk of metastasis (class 2A) had 3 year metastasis-free survival rates of 91.4% and 80.6%, respectively. Patients with the highest risk of metastasis (class 2B) had only a 44.0% chance of being free of metastasis after 3 years from the diagnosis; in this subgroup of patients, this test achieved a 60% positive predictive value [24]. In addition to genetic mutations and mutational patterns, alterations in the expression of genetic code may have a role in predicting the risk of CSCC metastasis. In an analysis of 48 RNA samples, despite the small sample size, several microRNAs were found to show differential expression between primary tumors which metastasized and those that did not [28]. Finally, in a proteomic-based study including 105 CSCC samples, half of which metastasized within 5 years from surgery, increased expression of two proteins, ANXA5 and DDOST, was significantly associated with development of metastasis, highlighting the potential role of proteomic analysis into risk stratification of patients with CSCC [29]. However, as of today, the impact of molecular and epigenetic characterization on prognosis and targeted treatments is not entirely clear, and it should not be routinely performed in everyday clinical practice, outside of translational studies (expert recommendation 4).

## 5. Precursors of Invasive CSCC

Invasive CSCC originates from a proliferation of keratinocytes as the ultimate step of a long-lasting process of intraepidermal dysplasia. Actinic keratoses (AKs) are considered precancerous lesions, while in situ forms of CSCC are called Bowen’s disease. In some classifications, AKs are considered as in situ lesions with different grades of dysplasia [30,31]. The rate of progression from AK to invasive CSCC is less than 1/1000 each year [32]. To date, there is no predictive model to identify which AK will transform into invasive CSCC and when this will happen, as well as identifying which is the risk of developing invasive CSCC in a patient with a previous diagnosis of CSCC in situ.

In a recent analysis including more than 1000 subjects with AK, the rate of patients developing an invasive CSCC was about 15% after a median follow-up of 1.8 years. Significant predictors of progression were presence of 4 to 9 AKs (HR 1.68, 95% CI 1.17–2.42), 10 or more AKs (HR 2.44, 95% CI 1.65–3.61), AK localization on the upper extremities (HR 0.75, 95% CI 0.52–1.08) or elsewhere except the head (HR 1.40, 95% CI 0.98–2.01), and coffee consumption (HR 0.92, 95% CI 0.84–1.01) [33]. The risk of developing invasive CSCC in patients with prior CSCC in situ was assessed in a cohort study of 88,754 patients with CSCC in situ [34]. As compared with the general population, patients with a diagnosis of CSCC in situ in the previous year had a 16-fold greater risk of developing invasive CSCC [34]. The results of these studies highlight the high burden of disease and social costs associated with AKs and CSCCs in situ, and the need to develop predictive models to optimize the resources for follow-up and secondary prevention programs.

Subjects with more than three AKs and/or with a previous diagnosis of CSCC in situ should undergo dermatologic follow-up due to the high risk of developing an invasive CSCC (expert recommendation 5). However, to date, no validated predictive model is available, and more efforts should be made regarding the development of an algorithm that could help clinicians to tailor follow-up based on the actual risk of progression. In fact, the driving molecular alterations underlying progression from AK to invasive and metastatic CSCC are poorly understood, and it is not clear if molecular characterization of AK may define their risk of cancerization. In a recent next-generation sequencing (NGS) analysis of invasive CSCC and CSCC precursors, precursor lesions had highly similar somatic genomic landscapes to CSCCs, including chromosomal gains of 3q involving SOX2, as well as highly recurrent mutations and/or loss of heterozygosity events affecting tumor suppressors TP53 and CDKN2A, supporting non-genetic drivers of invasiveness [35]. The role of epigenetic deregulation into the prediction of AK cancerization is also not completely understood. In a recent study, the methylation signature of AKs appeared to be similar to that of mCSCC, while it was different from that of initial invasive carcinomas and high-risk non-metastatic carcinomas [23]. To date, molecular and epigenetic profiling of AKs is not recommended in everyday clinical practice, outside of translational studies (expert recommendation 6).

## 6. Clinical Diagnosis and Secondary Prevention

CSCC most commonly arises in chronically sun-exposed anatomical sites (such as head and neck, dorsum of the hands, forearms) and may have heterogeneous clinical presentations depending on location, skin type, tumor size, differentiation, and pigmentation. Differential diagnosis between hyperplastic/hyperkeratotic AK or CSCC in situ and minimally invasive CSCC is not always easy. Early CSCCs usually present as small flesh-colored plaques or papules, with or without a scaly/hyperkeratotic surface, usually with usually some induration at palpation. Over time, they tend to enlargement and ulceration. CSCC can be pigmented, presenting a light to dark brown color. White circles, keratin, and blood spots are dermoscopic features that are helpful to differentiate CSCC and keratoacanthoma from other raised non-pigmented skin lesions [36]. Dermoscopic structures associated with abnormal keratinization to the hair follicle and adnexal structures correlate with well-differentiated tumors [36,37,38].

Some dermoscopic features are associated with the sequential phases of progression form an AK to an invasive CSCC. Red pseudonetwork is associated with AK, whereas dotted/glomerular vessels, diffuse yellow opaque scales, and microerosions are more frequently associated with CSCC in situ. Hairpin vessels, linear-irregular vessels, targetoid hair follicles, white structureless areas, a central mass of keratin, and ulceration are more prevalent in invasive CSCC. Patterns observed in CSCC were also found among keratoacanthomas [38].

Despite there being no large studies validating dermoscopy specifically for the diagnosis of CSCC, strong evidence supports the effectiveness of dermoscopy for the diagnosis of melanoma and other skin diseases, highlighting the important role of this technique in the overall differential diagnosis of skin tumors [36,39,40]. Therefore, the use of dermoscopy is recommended as an adjunct to visual inspection for the secondary prevention of keratinocyte cancers and melanoma (expert recommendation 7).

In addition to dermoscopy, other non-invasive techniques have been used, although in limited case series. In vivo reflectance confocal microscopy is a non-invasive diagnostic tool that generates characteristics correlating with histopathologic features, such as parakeratosis, atypical keratinocytes, and vascular alterations. In pigmented AKs, the main reflectance confocal microscopy features are the presence of epidermal changes (atypical keratinocytes, parakeratosis, scaling); increased epidermal thickness; bright, small, dermal papillae with enlarged interpapillary space; and intraepidermal dendritic cells referable to Langerhans cells [41].

Insufficient data are available to draw conclusion on the efficacy of confocal microscopy for the diagnosis of CSCC, and it may not be recommended for a routine diagnostic use for CSCC at this point in time. However, reflectance confocal microscopy may be employed in selected patients for the differential diagnosis of complex lesions, especially in the head and neck area (expert recommendation 8).

## 7. Histopathological Diagnosis

An excisional biopsy should be performed in all clinically suspected CSCCs for histological confirmation. An incisional or punch biopsy may be performed, depending on the size and location of the tumor.

CSCCs are malignancies of epidermal keratinocytes exhibiting varying degrees of differentiation that partially recapitulate the cytology of squamous cells of the epidermal stratum spinosum. Immunohistochemistry exhibits positivity for P63, p40, EMA, cytokeratins CK5/6, MNF116, and 34 beta E12, while BerEp4 is negative, in contrast to basal cell carcinoma. Occasionally, CSCCs show morphological evidence of neuroendocrine differentiation. As for differential diagnosis, well-differentiated CSCC must be distinguished from pseudoepitheliomatous hyperplasia, syringometaplasia, cells traumatically introduced into scars, perineural hyperplasia, endophytic keratoses, warts, and squamoid adnexal tumours. Poorly differentiated SCC must be differentiated from other tumors, often requiring immunohistochemistry [14]. 

The classification of CSCC according to the WHO, and the differences between the third and fourth editions, is shown in Table 1 [14]. Acantholytic squamous cell carcinoma, spindle cell squamous cell carcinoma, adenosquamous carcinoma, and squamous cell carcinoma with sarcomatoid differentiation are high-risk histological subtypes. Among the strongest pathological risk factors, perineural invasion (PNI) is defined as the presence of tumor cells in the nerve sheath of a nerve lying deeper than the dermis or measuring 0.1 mm or larger in caliber or presenting with clinical or radiographic involvement of named nerves without skull base invasion. It is more frequent in poorly differentiated (spindle cell) tumors with desmoplasia. Small-caliber perineural invasion has no independent prognostic impact [36]. The depth of invasion (measured in mm from the granular layer of adjacent normal epidermis to the base of the tumor) and level of invasion correlate with the metastatic potential of the tumor. “Deep invasion” is defined as a thickness >6 mm or the invasion of tumor cells beyond the subcutaneous fat (fascia, muscle tissue, perichondrium, periosteum, etc.) [42].

Keratoacanthoma (syn.: well differentiated CSCC (keratoacanthoma type) or cheratoacantoma-like CSCC) is a rapidly growing squamo-proliferative tumor that may spontaneously regress. It is histologically indistinguishable from and likely to be a variant of well-differentiated cutaneous invasive squamous cell carcinoma with distinct clinical behavior. In the early proliferative stage, the tumor is symmetrical and composed of invaginations of interconnecting follicular infundibular/isthmus-type squamous epithelium. The squamous cells contain pale, glassy, eosinophilic cytoplasm and show abrupt (trichilemmal) keratinization devoid of an intervening granular cell layer; rare mitoses; dense mixed neutrophilic, lymphocytic, and eosinophilic infiltrate; and intraepithelial microabscesses. The mature stage displays a typical crateriform architecture. Well-differentiated squamous lobules showing follicular differentiation with trichelemmal keratinization surround a central keratin core. Epidermal lipping is present on both sides of the keratin core. The regressing stage retains a central invagination and crateriform appearance, although the tumor becomes thinner and flattened with fewer squamous lobules and horn cysts around the crater. A dense mixed inflammatory infiltrate and dermal scar-like fibrosis are present at the base [14].

Since clinical data are part of the prognostic staging, the following minimum clinical information should be recorded preoperatively and provided to the pathologist (expert recommendation 9):

- Age, sex;
- Anatomic site, association with actinic keratosis (field cancerization);
- Maximum tumor diameter (in mm, evaluated prior to surgery);
- Primitive tumor or relapse;
- Immunosuppression (specify: recipient of an organ transplant, previous diagnosis with chronic leukemia) and other comorbidities (previous RT, burns, chronic inflammation or scars, etc.);
- Lifestyle (smoking, alcohol consumption);
- Skin specimen orientation/labeled margins if necessary.

The final histopathological report should include histological risk factors that are relevant for the staging and prognosis of CSCC. The following minimum information should be provided in the histology report by the pathologist (expert recommendation 10):

- Histotype (WHO 4th Edition, 2018);
- Grade of differentiation;
- Depth of invasion (the maximum vertical tumor thickness is measured in millimeters, from the granular layer of adjacent normal epidermis to the base of the tumor);
- Clark level of invasion;
- Desmoplasia;
- Perineural invasion (PNI);
- Lymphovascular invasion (LVI);
- Invasion of fascia, muscle, or bone;
- Association with precursor lesions (actinic keratosis/actinic cheilitis) or de novo;
- HPV infection (only for selected sites);
- Margin status;
- AJCC TNM stage (8th Edition).

Pathological margins should be reported as proposed by the Royal College of Pathologists (Table 2) (expert recommendation 11) [43].

## 8. Prognostic Factors for High-Risk CSCC

Only few CSCCs are associated with a high-risk of local or distant relapse; therefore, it is particularly relevant to identify high-risk tumors among all other low-risk CSCCs for the proper diagnostic and therapeutic management, in addition to a tailored follow-up. Risk factors may be classified as tumor- and patient-related; high-risk prognostic factors identified in recent staging systems and guidelines are summarized in Table 3 [42,44,45,46]. The variability of the characteristics included in each classification and guideline reflects the variable results obtained in clinical studies, mostly with a retrospective design and with a limited number of patients [17,42,44,45,46,47,48,49,50,51,52]. Moreover, the impact of each individual factor on the overall risk is not completely understood, and there is no clear consensus on how many risk factors define a high-risk case. Finally, the binary classification into low- and high-risk cases may be an over-simplification of a more complex clinical and pathological situation, with the presence of intermediate-risk cases of difficult definition. In Table 4, risk ratios for CSCC recurrence, metastasis, and disease-specific death are reported for some risk factors included for assessment in a recently published meta-analysis [52], and may be of help for the clinician for risk prognostication.

The definition of high-risk CSCC is highly variable, and the impact on the risk of relapse and death is not completely understood for each risk factor included in the different staging systems and classifications (see Table 3 and Table 4). Primary CSCCs showing at least one risk factor are too many to be all defined as high-risk lesions (given the overall low risk of relapse), and tumors that have in fact low risk of recurrence may be over-treated and subjected to exaggerated imaging follow-up. Therefore, on the basis of the current evidence and expert opinions, a CSCC may be defined as high-risk and should be managed in a multidisciplinary setting if one of the following conditions apply (expert recommendation 12):

- Maximum tumor diameter > 20 mm or Breslow thickness > 6 mm or Invasion beyond subcutaneous fat with or without any other risk factors;
- 1 patient-related + 1 tumor-related clinical/radiological + 1 tumor-related pathological risk factors;
- 3 or more tumor-related risk factors with or without patient-related risk factors.

## 9. Surgical Treatment

Surgery is the primary treatment of CSCC, regardless the age-group of the patient and anatomic location of the lesion [53]. Surgical excision alone grants successful treatment of most CSCCs, with cure rates over 90% [15]. The large majority of CSCCs may be treated with conventional surgery with safety margins, while in selected cases, micrographically controlled surgery may be offered, especially for complex lesions in the head and neck area [49,54,55].

Negative pathologic margins are necessary to minimize the risk of relapse [44]. Evaluation of margins on intraoperative may be associated with a high rate of false negatives [56], and therefore further confirmation with paraffin-embedded sections is always required. Excision margins should be adapted to the risk of recurrence, as defined by tumor- and patient-related risk factors [44,57] (Table 5). The main tumor-related risk factors include: tumor diameter >2 cm, high-risk anatomical sites, thickness >6 mm or invasion beyond subcutaneous fat, PNI, poor differentiation, high-risk histological subtypes, recurrent tumor. The main patient-related risk factors include: immunosuppression, previous RT or chronic inflammatory process, neurological symptoms.

A margin of 4 mm achieved cure rates greater than 95% in case of low-risk (<2 cm) CSCC in prospective studies [58,59]. However, tumor diameter is not the only factor of tumor aggressiveness, and additional pathological features and patient-related characteristics may increase the risk of margin involvement despite small tumor dimensions [60]. Therefore, there is no complete concordance between guidelines, clinical recommendations, and consensus statements on how the minimum safety margins should be for low-risk CSCC [44,45]. The minimum clinical safety margins for low-risk CSCC should be at least 4 mm (expert recommendation 13).

There is no complete concordance between guidelines, clinical recommendations, and consensus statements on how the minimum safety margins should be for high-risk CSCC [40,44,45]. The minimum clinical safety margins for high-risk CSCC should be at least 6 mm; a narrower margin is acceptable on body sites at high morphofunctional impact and/or with limited tissue availability to avoid excessive morbidity (expert recommendation 14). Micrographically controlled surgery may be offered in case of high-risk or recurrent CSCC after a multidisciplinary discussion (expert recommendation 15). In case of tumors requiring extensive surgical excisions, the management should be entrusted to a multidisciplinary team, including surgeons with appropriate expertise in all kinds of reconstructive procedures.

## 10. Management of CSCC on Field Cancerization

**Figure 1:**
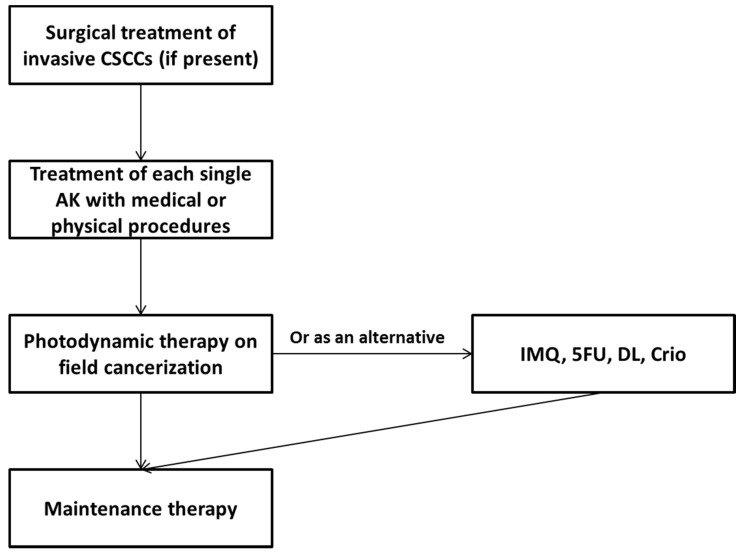
Algorithm of management of patients with actinic keratosis. Maintenance therapy may include agents such as nicotinamide, polypodium, and photoliasis. Abbreviations: 5FU: 5-fluororacil; AK: actinic keratosis; Crio: cryotherapy; CSCC: cutaneous squamous cell carcinoma; DL: day light photodynamic therapy; IMQ: imiquimod.

Despite the rate of progression of each AK being very low, in patients with a high number of AKs, the overall risk may be high [32,33], and a treatment on field cancerization may be indicated (expert recommendation 16). The management of field cancerization should start with an accurate inspection to exclude the presence of CSCC (invasive or in situ); if detected, they must be properly treated before the field cancerization. Then, each single AK should be treated with a medical or physical procedure (such as cryotherapy). Treatment on field cancerization relies on several strategies with different mechanisms of action, such as photodynamic therapy, 5-fluorouracil cream, imiquimod cream, diclofenac sodium gel, salicylic acid, and retinoids (Figure 1).

Due to the high risk of relapse after field cancerization treatment, a maintenance treatment (nicotinamide, polypodium, photoliasis) may be offered to patients (expert recommendation 17). Meta-analysis and review data are limited to high-risk populations, and clinical trials are needed to unravel their clinical value as well as their long-term safety.

Finally, a non-surgical treatment on field cancerization after surgery of CSCC of the head and neck area (or any other anatomical region with field cancerization) may be offered to reduce the risk of incidence of additional invasive tumors (expert recommendation 18). In fact, patients with a previous diagnosis of CSCC have a very high risk of developing new primary CSCCs and other skin cancers (almost 50% at 5 years) [52]. Non-surgical treatments include photodynamic therapy, 5-fluorouracil cream, imiquimod cream, diclofenac sodium gel, salicylic acid, and retinoids, and are frequently used for the management of AKs. However, evidence on this setting is scarce and clinical trials should be designed to address the efficacy of this approach.

## 11. Sentinel Lymph Node Biopsy

Sentinel lymph node biopsy (SNLB) may have an impact into regional metastases diagnosis anticipation and early treatment, in addition to better prognosis stratification and patient’s selection for adjuvant clinical trials. However, the evidence on the role of SNLB and management of regional nodal disease in patients with CSCC is very limited and relies on retrospective case series only, and the appropriate clinical setting for the application of SLNB is not well characterized. For these reasons, SNLB is not currently recommended in most international guidelines.

In a meta-analysis of 19 studies for a total of 130 patients with CSCC who underwent SLNB, a positive sentinel lymph node was identified in 12.3% of all patients, with a false-negative rate of 2.6%. In all cases of positive sentinel lymph node, the diameter of the primary CSCC was greater than 2 cm. According to AJCC criteria, positive sentinel lymph nodes were found in 0 of 9 T1 lesions (0%), 13 of 116 T2 lesions (11.2%), and 3 of 5 T4 lesions (60.0%); no T3 lesions were identified [61]. In a more recent systematic review, the rate of positive SLNB was 13.9% (32 of 231 patients), with a false-negative rate of 4.6% (10 of 215 patients) [62]. In the most recent systematic review on this topic, the proportion of patients with CSCC and positive SLNB was 8% [56], according 23 studies and 566 patients analyzed, lower than that observed in previous analyses [61,62]. No studies reporting on predictors of sentinel lymph node involvement or on the prognostic utility positive SLNB following adjustment for confounders were found, and criteria for recommending SLNB varied considerably from study to study [63]. None of the studies were large enough to reliably identify predictors of SLN positivity [63].

The appropriate clinical setting for the application of SLNB in patients with CSCC is not well characterized, and there is no sufficient evidence on the prognostic value of SNLB to recommend such procedure in patients with CSCC in everyday clinical practice; however, the procedure may be offered after multidisciplinary consultation in selected patients, preferably in the context of clinical studies (expert recommendation 19). More efforts should be addressed into identifying predictors of sentinel lymph node involvement for proper patient selection.

## 12. Staging Systems and Radiologic Imaging

The staging systems most commonly used for CSCC are the AJCC 8th edition and the BWH T classification systems. The AJCC 8th edition was designed to classify patients with CSCC of the head and neck region only. Other invasive CSCC (excluding perianal, vulva, and penis) may be staged using the UICC 2017 staging system [64]. Notably, in the AJCC classification, grade of differentiation is not included among the factors identifying high-risk cases, while invasion beyond subcutaneous fat and PNI upstage the tumor to T3, and bone invasion upstages to T4 [42]. Major limitations of the AJCC 8th edition staging system include: some T2 tumors may be associated with poor outcomes, especially if poorly differentiated; very few tumors meet the criteria for T4 subgroup, which is used rarely; stage III group is highly heterogeneous, and includes both patients with primary CSCC only and patients with lymph node metastases. The BWH classification system (Table 6) is based on a multivariate analysis of a retrospective cohort study and provides a quantifiable risk value according to the number of risk factors (poor differentiation, PNI, invasion beyond the subcutaneous tissue, and diameter ≥ 2 cm).

In a study evaluating the impact of radiologic imaging on the management and outcomes of high-stage CSCC, patients who received no imaging were at higher risk of developing nodal metastases, local recurrence, and death from disease compared to the imaging group. Imaging was associated with a lower risk for recurrence and death after adjusting for Brigham and Women’s Hospital T stage, sex, and anatomical location [65]. These results support the use of radiologic imaging in the management of high-risk CSCC. The choice of imaging modality is related to the clinical context. In particular, ultrasound should be used in case of operable high-risk tumors without palpable nodes for the diagnosis of non-palpable lymph node metastases; CT scan and MRI should be employed to assess tumor extension in locally advanced tumors, and whole-body CT or PET/CT for locally advanced tumors to assess potential distant metastases (expert recommendation 20).

## 13. Follow-Up

Patients with CSCC should be followed up for the early diagnosis of new primary skin cancers and for CSCC recurrences. In a cohort of 1426 patients with CSCC, the risk of new primary CSCC or other skin cancer was 42% at 5 years and 69% at 10 years; overall risk of local and locoregional relapse were 4.3% and 1.3%, respectively. Notably, 96% of all recurrences occurred within 2 years [52]. Risk of relapse is significantly higher in patients who are immunocompromised [66,67].

**Figure 2:**
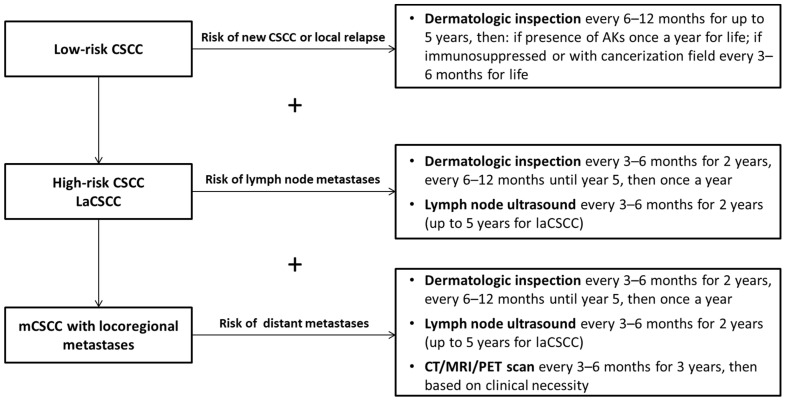
Algorithm for the follow-up of patients with cutaneous squamous cell carcinoma. Dermatologic inspection should always include lymph node palpation. Clinical evaluation and imaging should be tailored to patient life expectancy and comorbidities. Additional exams may be necessary according to symptoms or clinical suspect. Abbreviations: AK: actinic keratosis; CSCC: cutaneous squamous cell carcinoma; laCSCC: locally advanced cutaneous squamous cell carcinoma; mCSCC: metastatic cutaneous squamous cell carcinoma.

There is no standardized follow-up schedule for patients with CSCC. In Figure 2, we present an algorithm for the follow-up of patients with CSCC that is based on the specific risks associated with each CSCC stage, from low-risk to metastatic. Dermatologic inspection should always include lymph node palpation. Visits and imaging should be tailored to patient life expectancy and comorbidities. Additional exams may be necessary, according to symptoms or clinical suspect.

## 14. Radiotherapy

No randomized trials comparing the outcomes of radiotherapy (RT) for the treatment of primary CSCC compared with other local therapy modalities have been conducted. Current evidence relies on observational studies. In a meta-analysis of 14 observational studies for a total of 1018 primary CSCCs, the local relapse with RT was 6.4%.

In cases of patients with primary CSCC who are not eligible for curative surgery, or when surgery is not a proper option due to inacceptable functional and aesthetic outcomes, definitive primary RT may be considered as a primary treatment after a careful multidisciplinary evaluation to assess the possibility of a systemic approach with anti-PD-1 immunotherapy (expert recommendation 21) [53].

Postoperative RT may be considered in case of positive margins if re-excision is not possible or contraindicated (expert recommendation 22) [44,45,68].

Adjuvant RT improved disease-free survival (DFS) and overall survival (OS) in a series of 349 patients with CSCC on the head and neck region with regional metastases or PNI [61]. Therefore, local adjuvant RT may be offered to patients with high-risk CSCC, particularly in case of PNI (expert recommendation 23) [44,45].

Adjuvant RT improved DFS and OS in a series of 349 patients with CSCC on the head and neck region with regional metastases or PNI [68]. A recent meta-analysis confirmed the improved DFS and OS of adjuvant RT; extra-capsular extension was associated with poorer OS [69]. Therefore, regional adjuvant radiotherapy may be offered after surgical excision of regional metastases, particularly in case of extra-capsular extension (expert recommendation 24).

## 15. Systemic Treatments for Advanced CSCC

Advanced CSCC include both laCSCC and mCSCC. Locally advanced CSCC may be defined as tumors not amenable to either surgery or radiotherapy with reasonable curative intent or long-lasting disease control. It includes a heterogeneous variety of tumors. To define a tumor as locally advanced, multiple tumor- and patient-related factors must be considered, preferably in a multidisciplinary context:

- Large tumor extension, bone invasion, or deep tissue infiltration;
- Multiple recurrences;
- Anatomical sites in which curative resection would result in unacceptable complications, morbidity, or deformity;
- Multiple tumors;
- Comorbidities, age, performance status;
- Anticipated patient’s quality of life;
- Patient’s preferences.

Metastatic CSCC includes cases with locoregional metastases (in-transit or lymph node metastases) and cases with distant metastases.

Currently, there is no chemotherapy approved for the treatment of advanced CSCC. Platinum agents (cisplatin and carboplatin), 5-fluorouracil, bleomycin, methotrexate, taxanes, capecitabine, doxorubicin, and gemcitabine are the chemotherapies most commonly used as a single agent or in polichemotherapy regimens for the treatment of CSCC, but data on these treatments mostly rely on retrospective studies with a small number of patients, and there is no standard of care [47,70,71]. Despite a high overall response rate with mono- and polichemotherapy, responses are mostly short-lived and do not lead to a curative effect [70]. In addition to the poor efficacy, chemotherapy regimens, especially with more agents used in combination, are associated with severe toxicities and are often contraindicated in patients with CSCC, who are mostly of old age and with comorbidities. In a large retrospective analysis, once diagnosed with laCSCC, most patients (59%) did not receive any systemic therapy due to anticipated poor efficacy and tolerability [47].

Elevated expression and, in a small subset of cases, somatic mutations of epidermal growth factor receptor (EGFR) have been demonstrated in advanced CSCC [72,73]. The EGFR inhibitor most frequently used for advanced CSCC is cetuximab, with response rates ranging from 28 to 48.5%, and PFS from 4.1 to 9 months (Table 7) [74,75].

Due to an etiopathogenesis largely relying on chronic UV radiation exposure, CSCC is among the tumors with the highest rate of somatic mutations, which are associated with increased response rates to immunotherapy [76]. Mutated proteins may in fact serve as neoantigens which can be recognized by the immune system [77]. Therefore, there was a strong biological rationale to testing anti-PD-1 immunotherapy in patients with advanced CSCC [16].

Cemiplimab is a fully human immunoglobulin G4 (IgG4) monoclonal antibody that blocks the interaction between the PD-1 receptor and its ligands PD-L1 and PD-L2, which are expressed not only by antigen-presenting cells but also by tumor cells and other tumor microenvironment cells. Since the engagement of PD-1 with its ligands results in inhibition of T cell function, cemiplimab, through blockade of PD-1 binding to PD-L1 and PD-L2, potentiates T cell responses, including anti-tumor responses.

After the encouraging results of a phase 1/2 trial [78], which led to the FDA and EMA approval of cemiplimab for the treatment of mCSCC or laCSCC not amenable to curative surgery or curative radiation, the efficacy and safety of cemiplimab in patients with mCSCC (nodal or distant) or laCSCC were further investigated in the EMPOWER-CSCC 1 clinical trial. In this phase 2, non-randomized, open-label, multi-center study, 193 patients with advanced CSCC who were not candidates for curative surgery or radiation were treated with either cemiplimab 3 mg/kg every 2 weeks (group 1 and 2 with mCSCC and laCSCC, respectively) or 350 mg every 3 weeks (group 3 with mCSCC). The latter is the currently approved regimen of cemiplimab. At the last update of the EMPOWER-CSCC-1 study, presented at ASCO Annual Meeting 2020 by Rischin et al., the pooled overall response rate for the three groups was 46.1%, with 16.1% of complete responses. The kinetics of tumor regression as fast, with most responses being observed at first tumor assessment for a median time of response of 2.1 months. Disease control rate was 72.5%. Both median PFS and OS were not reached. Estimated median PFS was 18.4 months, while estimated OS at 2 years was 73.3% [79]. Patients who received cemiplimab for laCSCC that recurred more than once after surgical excision had less than half the chance of responding to immunotherapy compared with patients who received front-line systemic therapy, highlighting the importance of proper patient selection for either front-line surgery or immunotherapy [80]. Clinical activity was observed regardless of tumor PD-L1 expression status. Notably, cemiplimab also had a significant impact on pain reduction and overall quality of life improvement [81]. The tolerability of cemiplimab is similar to that reported for other anti-PD-1 drugs, such as nivolumab and pembrolizumab, with less than 10% of patients interrupting treatment due to adverse events [78].

Despite the absence of direct comparison, on the basis of the results of the phase 1 and 2 studies, cemiplimab compares favorably to chemotherapy and cetuximab, and the conduction of a randomized phase 3 trial would be unethical. Therefore, treatment with cemiplimab should be offered to patients with CSCC when curative surgery or curative radiotherapy is not appropriate (expert recommendation 25). Chemotherapy and/or cetuximab may be considered as second-line systemic treatments (expert recommendation 26).

Patients who received front-line immunotherapy for laCSCC had more than twice the chance of achieving a response compared to patients who had two or more surgeries [80]. Therefore, it is of great importance to properly select patients with CSCC and numerous tumor- and patient-related risk factors to receive either front-line surgery or immunotherapy, according to a multidisciplinary assessment of the risk, benefits, and costs of both interventions and their anticipated impact on long-term disease control and survival. Therefore, for patients with CSCC and numerous risk factors, cemiplimab may be offered as a front-line treatment compared to surgery if it is anticipated, after a careful multidisciplinary evaluation, that it may achieve the highest chances of long-term outcomes (expert recommendation 27).

## 16. Special Populations

Median age of patients included in clinical trials was over 70 years and ranged up to 96 years. However, on the basis of the exclusion criteria of such trials, patients with autoimmune disease requiring systemic immunosuppression, with a history of solid organ transplant, with an HIV/HBV/HCV infection, and with chronic lymphocytic leukemia, could not be enrolled in the studies. Since immunosuppression is one of the strongest risk factors for CSCC, in everyday clinical practice, patients with advanced CSCC often have one or more of these comorbidities.

Retrospective analyses of patients with advanced cancer and autoimmune disease who received anti-PD-1/PD-L1 immunotherapy showed similar clinical activity than that observed in patients without autoimmune comorbidities. However, an exacerbation of their autoimmune condition was observed in up to 50% of patients. There was no discrepancy in response rate between those who did and did not experience an exacerbation of their condition [82,83]. Therefore, patients with advanced CSCC and an autoimmune disease should be offered treatment with cemiplimab after a careful multidisciplinary assessment and a strict follow-up to detect possible exacerbation of the pre-existing autoimmune condition; a thorough discussion with the patient on the risks of symptoms exacerbation and other potential risks and benefits of immunotherapy is necessary before beginning treatment (expert recommendation 28).

Although only a small number of cases have been reported, immune checkpoint inhibitors have been used in patients who received organ transplantation. In a recently published systematic review, an organ rejection rate as high as 37% was reported among 57 transplanted patients who were treated with immune checkpoint inhibitors for advanced cancers, and 14% of them died due to graft rejection. Patients with kidney transplants had the highest rejection rate (40%), followed by liver (35%) and heart (20%) transplants. Anti-PD-1 drugs achieved an overall response rate of 30–40% [84]. Therefore, patients for whom there is no valid alternative for the treatment of advanced CSCC should be offered treatment with cemiplimab, although a thorough discussion with the patient on the risk of graft rejection and other potential risks and benefits of immunotherapy is necessary before beginning treatment (expert recommendation 29).

Patients with HIV infection have been excluded from most trials investigating immune checkpoint inhibitors. Despite that, accumulating evidence shows that immunotherapy with anti-PD-1/PD-L1 agents is safe and maintains clinical activity. In a retrospective analysis of 44 patients with HIV infection who received anti-PD-1/PD-L1 drugs for the treatment of advanced cancers, the administration of immunotherapy did not impact negatively on the viral control and had clinical activity [85]. In a study evaluating the safety of pembrolizumab in patients with HIV, median CD4+ T-cell counts tended to increase over time, and HIV remained suppressed in all patients [86]. Therefore, treatment with cemiplimab should be offered to patients with advanced CSCC and HIV infection, after a careful multidisciplinary assessment and with a strict follow-up for possible effects on the viral control; a thorough discussion with the patient on the potential risks and benefits of immunotherapy is necessary before beginning treatment (expert recommendation 30).

Retrospective data show that immune checkpoint inhibitor treatment has equivalent therapeutic efficacy in patients with advanced melanoma and chronic lymphocytic leukemia [87,88,89]. Patients with advanced CSCC and chronic lymphocytic leukemia should be offered treatment with cemiplimab after a careful multidisciplinary assessment and a strict hematological follow-up; a thorough discussion with the patient on the potential risks and benefits of immunotherapy is necessary before beginning treatment (expert recommendation 31).

## 17. Conclusions

A summary of clinical recommendations regarding the treatments and follow-up for CSCC is provided in Table 8. Although anti-PD-1 drugs demonstrated great anti-tumor efficacy in clinical trials and in the real world [90], advanced CSCC is still a major therapeutic challenge and requires a multidisciplinary approach to be effectively managed, especially in case of patients with relative contraindications to immunotherapy. Several clinical trials are ongoing to investigate the activity of anti-PD-1 agents in the adjuvant (NCT03969004, NCT03833167) and neoadjuvant (NCT04632433, NCT04808999, NCT04315701, NCT04428671) settings. If the results shown by preliminary reports [91] are confirmed in larger series, the results of these studies will provide further advances in this challenging field.

## Figures and Tables

**Table 1:** WHO classification of skin tumors: differences between third and fourth editions.

WHO Classification 3rd Edition		WHO Classification 4th Edition	
Keratinocytic Tumours		Keratinocytic/Epidermal Tumors/Carcinomas	
Squamous Cell Carcinoma	8070/3	Squamous Cell Carcinoma, NOS	8070/3
Acantholytic squamous cell carcinoma	8075/3	Keratoacanthoma	8071/3
Spindle-cell squamous cell carcinoma	8074/3	Acantholytic squamous cell carcinoma *	8075/3
Verrucous squamous cell carcinoma	8051/3	Spindle cell squamous cell carcinoma *	8074/3
Pseudovascular squamous cell carcinoma	8075/3	Adenosquamous carcinoma *	8051/3
Adenosquamous carcinoma	8560/3	Clear cell squamous cell carcinoma	8560/3
		**Other (uncommon) variants**	
Bowen disease	8081/2	Squamous cell carcinoma with sarcomatoid differentiation *	8074/3
Bowenoid papulosis		Lymphoepithelioma-like carcinoma	8082/3
		Pseudovascular squamous cell carcinoma	8074/3
		Squamous cell carcinoma with osteoclast-like giant cells	8035/3
		Squamous cell carcinoma in situ (Bowen disease)	8070/2

* High risk.

**Table 2:** Template for reporting pathological margins as proposed by the Royal College of Pathologists.

Margins	Involved	Not Involved			Uncertain	Not Applicable
		<1 mm	1–5 mm	>5 mm		
Peripheral	□	□	□	□	□	□
Deep	□	□	□	□	□	□

**Table 3:** Summary of high-risk factors for primary squamous cell carcinoma according to recent staging systems and guidelines.

High-Risk Factors	AJCC 8th ed. Classification	BWH Classification	NCCN Guidelines	EADO Guidelines
**Patient-related risk factors**				
Immunosuppression	Not included	Not included	Yes	Yes
Site of prior RT or chronic inflammation	Not included	Not included	Yes	Not included
Neurological symptoms/symptomatic PNI	Yes	Not included	Yes	Yes
**Tumor-related clinical risk factors**				
Large clinical diameter	>2 cm	≥2 cm	≥2 cm area L *	>2 cm
			≥1 cm area M *	
			Any for area H *	
Anatomical location of primary tumor	Not included	Not included	Area H	Ear, lip, temple
Poorly defined tumor borders	Not included	Not included	Yes	Not included
Rapidly growing tumor	Not included	Not included	Yes	Not included
Recurrent tumor	Not included	Not included	Yes	Not included
**Tumor-related radiological risk factors**				
Bone involvement	Yes	Yes	Not included	Yes
Perineural invasion on imaging	Yes	Yes	Not included	Yes
**Tumor-related histological risk factors**				
Breslow thickness > 6 mm	Yes	Yes	Yes	Yes
Poor differentiation	Not included	Yes	Yes	Yes
High-risk histological subtype	Not included	Not included	Yes	Yes
Perineural invasion	Yes	Yes	Yes	Yes
Lymphatic or vascular invasion	Not included	Not included	Yes	Not included
Invasion beyond subcutaneous fat	Yes	Yes	Yes	Yes

Abbreviations: BWH: Brigham and Women’s Hospital; PNI: perineural invasion; RT: radiotherapy. * Area L: trunk and extremities (excluding hands, nail units, pretibial, ankles, feet); Area H: central face, eyelids, eyebrows, periorbital, nose, lips (cutaneous and vermillion), chin, mandible, preauricular and postauricular skin/sulci, temple, ear, genitalia, hands, and feet; Area M: cheeks, forehead, scalp, neck, and pretibial.

**Table 4:** Risk factors for cutaneous squamous cell carcinoma recurrence, metastasis, and disease-specific death.

High-Risk Factors	RR for Local Relapse	RR for Locoregional Relapse	RR for Disease-Specific Death
**Tumor-related (clinical)**			
Maximum tumor diameter > 20 mm	3.22	6.15	19.10
Temple, ear, or lip location	3.20	2.82	4.67
**Tumor-related (pathological)**			
Breslow thickness > 6 mm	7.13	6.93	-
Invasion beyond subcutaneous fat	7.61	11.21	4.49
Poor differentiation	2.66	4.98	5.65
PNI	4.30	2.95	4.06
**Patient-related**			
Immunosuppression	1.51	1.59	0.35

Abbreviations: NA: not available; PNI: perineural invasion; RR: risk ratio.

**Table 5:** Minimum safety margins recommended by the NCCN, EDF-EADO-EORTC, and AIOM for the surgical treatment of low- and high-risk cutaneous squamous cell carcinoma.

	NCCN	EDF-EADO-EORTC	AIOM
**Low-risk**	4–6 mm	5 mm	4 mm
**High-risk**	6–9 mm	10 mm	6 mm

**Table 6:** T classification for cutaneous squamous cell carcinoma according to Brigham and Women’s Hospital staging system.

	T Category
**TX**	Primary tumor cannot be identified
**T0**	Carcinoma in situ
**T1**	Primary tumor without risk factors
**T2a**	Primary tumor with 1 risk factor
**T2b**	Primary tumor with 2–3 risk factors
**T3**	Primary tumor with 4 risk factors or bone invasion
**Risk factors**	• Tumor diameter ≥ 2 cm • Poor differentiation • Perineural invasion • Tumor invasion beyond subcutaneous fat

**Table 7:** Summary of results with chemotherapy and cetuximab for the treatment of advanced cutaneous squamous cell carcinoma (CSCC).

	Overall Response Rate	Duration of Response	Progression-Free Survival
**Chemotherapy** [63]	44%	Not available	5.5 months
**Cetuximab** [67,68]	28–48.5%	5 months	4.1–9 months

**Table 8:** Summary of clinical recommendations.

Topic	Clinical Recommendations
**Epidemiology and risk factors**	All CSCC cases, including in situ lesions, should be included in cancer registries, with both tumor- and patient-related information, to ensure consistent collection of information and to facilitate analysis of data. To ensure homogeneity of data collection, all pathologists should adhere to the WHO classification of Skin Tumours 4th edition. CSCC should be considered as an occupational disease in a subset of outside workers at high risk of developing skin cancer and be recorded in specific registries to gain access to specific welfare benefits.
**Pathogenesis and precursors of invasive CSCC**	4. As of today, the impact of molecular and epigenetic characterization on prognosis and targeted treatments is not clear, and it should not be routinely performed in clinical practice, outside of translational studies. 5. Subjects with more than three AKs and/or with a previous diagnosis of CSCC in situ should undergo dermatologic follow-up due to the high risk of developing an invasive CSCC. 6. To date, molecular and epigenetic profiling of AKs is not recommended in everyday clinical practice, outside of translational studies.
**Diagnosis**	7. Dermoscopy is a valuable tool for the differential diagnosis of skin lesions and should also be employed as an adjunct to visual inspection of a suspicious skin lesion following a thorough anamnesis, including assessment of risk factors for keratinocyte cancer and melanoma. 8. Reflectance confocal microscopy may be employed in selected patients for the differential diagnosis of complex lesions, especially in the head and neck area. 9. The following minimum clinical information should be recorded preoperatively and provided to the pathologist: age, sex, anatomic site, association with actinic keratosis (field cancerization), maximum tumor diameter (in mm, evaluated prior to surgery), primitive tumor or relapse, immunosuppression (specify: recipient of an organ transplant, previous diagnosis with chronic leukemia) and other comorbidities (previous RT, burns, chronic inflammation or scars, etc.), lifestyle (smoking, alcohol consumption), and skin specimen orientation/labeled margins if necessary. 10. The following minimum information should be provided in the histology report by the pathologist: histotype (WHO 4th Edition, 2018); grade of differentiation; depth of invasion (the maximum vertical tumor thickness is measured in millimeters, from the granular layer of adjacent normal epidermis to the base of the tumor); Clark level of invasion; desmoplasia; perineural invasion (PNI); lymphovascular invasion (LVI); invasion of fascia, muscle, or bone; association with precursor lesions (actinic keratosis/actinic cheilitis) or de novo; HPV infection (only for selected sites); margin status; and AJCC TNM stage (8th Edition). 11. Pathological margins should be reported as proposed by the Royal College of Pathologists (see Table 2).
**Prognostic factors**	12. A CSCC may be defined as high-risk and should be managed in a multidisciplinary setting if one of the following conditions apply: - maximum tumor diameter > 20 mm or Breslow thickness > 6 mm or invasion beyond subcutaneous fat with or without any other risk factors; - 1 patient-related + 1 tumor-related clinical/radiological + 1 tumor-related pathological risk factors; - 3 or more tumor-related risk factors with or without patient-related risk factors.
**Surgical and non-surgical treatments**	13. The minimum clinical safety margins for low-risk CSCC should be at least 4 mm. 14. The minimum clinical safety margins for high-risk CSCC should be at least 6 mm. 15. Micrographically controlled surgery may be offered in case of high-risk or recurrent CSCC after a multidisciplinary discussion. 16. In patients with a high number of AKs, a treatment on field cancerization may be indicated. 17. A maintenance treatment with nicotinamide, polypodium, or photoliasis may be offered to patients after a treatment on field cancerization due to the high risk of relapse. 18. A non-surgical treatment on field cancerization after surgery of CSCC of the head and neck area (or any other anatomical region with field cancerization) may be offered to reduce the risk of incidence of additional invasive tumors. 19. SNLB is not recommended in everyday clinical practice; however, the procedure may be offered after multidisciplinary consultation in selected patients, preferably in the context of clinical studies.
**Radiologic imaging and follow-up**	20. The choice of imaging modality is related to the clinical context. In particular, ultrasound should be used in case of operable high-risk tumors without palpable nodes for the diagnosis of non-palpable lymph node metastases; CT scan and MRI should be employed to assess tumor extension in locally advanced tumors; whole body CT or PET/CT for locally advanced tumors to assess potential distant metastases.
**Radiotherapy**	21. In case of patients with primary CSCC who are not eligible for curative surgery, or when surgery is not a proper option due to inacceptable functional and aesthetic outcomes, definitive primary RT may be considered as a primary treatment after a careful multidisciplinary evaluation to assess the possibility of a systemic approach with anti-PD-1. 22. Postoperative RT may be considered in case of positive margins if re-excision is not possible or contraindicated. 23. Local adjuvant RT may be offered to patients with high-risk CSCC, particularly in the case of PNI. 24. Regional adjuvant may be offered after surgical excision of regional metastases, particularly in the case of extra-capsular extension.
**Systemic treatments**	25. Treatment with cemiplimab should be offered to patients with CSCC when curative surgery or curative radiotherapy is not appropriated. 26. Chemotherapy and/or cetuximab may be considered as second-line systemic treatments. 27. For patients with CSCC and numerous risk factors, cemiplimab may be offered as a front-line treatment compared to surgery if it is anticipated, after a careful multidisciplinary evaluation, that it may achieve the highest chances of long-term outcomes. 28. Patients with advanced CSCC and an autoimmune disease should be offered treatment with cemiplimab after a careful multidisciplinary assessment and a strict follow-up to detect possible exacerbation of the pre-existing autoimmune condition. 29. Anti-PD-1 therapy may be offered to post-transplant patients with advanced CSCC if there is no valid alternative treatment, after a careful multidisciplinary assessment and with caution due to the risk of graft rejection. 30. Treatment with cemiplimab should be offered to patients with advanced CSCC and HIV infection, after a careful multidisciplinary assessment and with a strict follow-up for possible effects on the viral control. 31. Patients with advanced CSCC and chronic lymphocytic leukemia should be offered treatment with cemiplimab after a careful multidisciplinary assessment and a strict hematological follow-up.

Abbreviations: AK: actinic keratosis; CSCC: cutaneous squamous cell carcinoma; CT: computed tomography; MRI: magnetic resonance imaging; PET: proton emission tomography; PNI: perineural invasion; RT: radiotherapy SNLB: sentinel lymph node biopsy; WHO: World Health Organization.
